# Intrinsic Regulation of Spatiotemporal Organization within the Suprachiasmatic Nucleus

**DOI:** 10.1371/journal.pone.0015869

**Published:** 2011-01-07

**Authors:** Jennifer A. Evans, Tanya L. Leise, Oscar Castanon-Cervantes, Alec J. Davidson

**Affiliations:** 1 Neuroscience Institute, Morehouse School of Medicine, Atlanta, Georgia, United States of America; 2 Department of Mathematics, Amherst College, Amherst, Massachusetts, United States of America; Vanderbilt University, United States of America

## Abstract

The mammalian pacemaker in the suprachiasmatic nucleus (SCN) contains a population of neural oscillators capable of sustaining cell-autonomous rhythms in gene expression and electrical firing. A critical question for understanding pacemaker function is how SCN oscillators are organized into a coherent tissue capable of coordinating circadian rhythms in behavior and physiology. Here we undertake a comprehensive analysis of oscillatory function across the SCN of the adult PER2::LUC mouse by developing a novel approach involving multi-position bioluminescence imaging and unbiased computational analyses. We demonstrate that there is phase heterogeneity across all three dimensions of the SCN that is intrinsically regulated and extrinsically modulated by light in a region-specific manner. By investigating the mechanistic bases of SCN phase heterogeneity, we show for the first time that phase differences are not systematically related to regional differences in period, waveform, amplitude, or brightness. Furthermore, phase differences are not related to regional differences in the expression of arginine vasopressin and vasoactive intestinal polypeptide, two key neuropeptides characterizing functionally distinct subdivisions of the SCN. The consistency of SCN spatiotemporal organization across individuals and across planes of section suggests that the precise phasing of oscillators is a robust feature of the pacemaker important for its function.

## Introduction

The mammalian circadian system controlling daily rhythms in behavior and physiology is an assembly of oscillators regulated by a central pacemaker within the suprachiasmatic nucleus (SCN) of the anterior hypothalamus [1]. The SCN displays robust electrical and biochemical rhythms that persist in individual neurons after synaptic communication is disrupted [2], [3]. Within cells of both the SCN and peripheral tissues, transcriptional-translational feedback loops regulate the rhythmic expression of clock genes and their protein products [4]. While the SCN and peripheral clocks appear to operate in a similar fashion at the molecular level, the SCN has unique network properties that synchronize oscillators within the population to form a functional pacemaker [5]. A critical question for understanding pacemaker function concerns how the numerous oscillators within the SCN are organized into a coherent tissue.

A common model of SCN anatomy divides the nucleus into two compartments that differ anatomically and functionally. In the mouse, a region often referred to as the “shell” is marked by a dense concentration of arginine vasopressin (AVP) neurons, whereas the “core” contains neurons immunoreactive for vasoactive intestinal polypeptide (VIP) and gastrin-releasing peptide [6]. Shell and core regions differ in the density of afferent terminals from the retina and other sources, photic responsiveness, and oscillatory function [3], . Further, shell and core regions generally project to overlapping structures in the brain, but differ in the pattern of efferent projections to local targets in the hypothalamus and thalamus [6], [13]. The shell and core model indicates that the SCN contains a diverse neural population organized into functional subunits, but it has been argued that dichotomous organizational schemas should be supplemented with topographical descriptions of pacemaker function [14]. Mapping SCN function, however, has been limited by its complex organization.

While intact within the network, neural oscillators display synchronized periods but do not necessarily exhibit identical phases. Neural oscillators within different SCN regions display peak clock gene/protein expression and electrical firing at different phases, and these phase differences are stable, non-random, and modulated by light [3], [12], [15]–[21]. However, the spatiotemporal organization of the SCN remains poorly understood due to discrepancies across studies in the description of regional phase differences. Previous studies have reported phase gradients spreading from dorsal to ventral regions [3], [19], ventral to dorsal regions [20], [21], lateral to medial regions [18], and caudal to rostral regions [15], [22]–[24]. These studies provide compelling evidence that spatially distinct SCN regions adopt different phases; however, little is known about the mechanistic bases of SCN phase heterogeneity and the degree to which regional phase differences are determined by light input, distinct oscillatory function, or neuropeptide content.

We have developed a novel approach for mapping oscillatory function within the SCN involving multi-position automated bioluminescence imaging and extensive computational analyses. Here we use these techniques to provide a comprehensive analysis of SCN phase heterogeneity and to investigate its mechanistic bases. Our results underscore the sophistication of SCN spatiotemporal organization, indicating that there is phase heterogeneity across all three dimensions of the pacemaker. Further, we present evidence that phase differences are not linearly related to regional differences in other oscillatory parameters or AVP and VIP content. Lastly, we demonstrate that SCN phase heterogeneity is not driven by light input but modulated by photic stimuli in a region-specific manner.

## Results

### The SCN displays robust regional phase differences under standard lighting conditions

**Figure 1:**
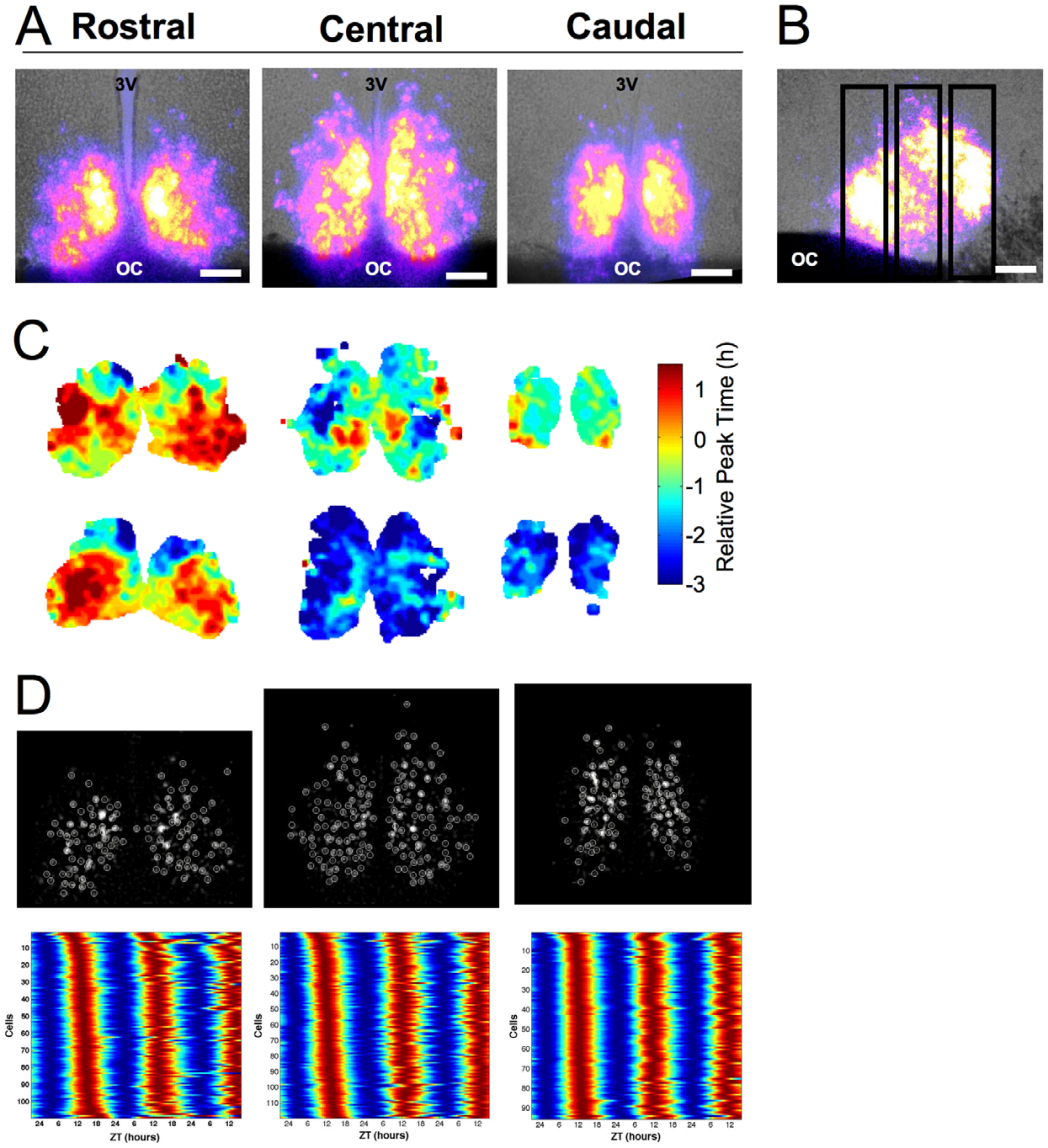
Mapping phase heterogeneity across the SCN. A. Representative coronal SCN slices (125µm) from a LD 12∶12 animal. Total bioluminescence over the first 24h pseudocolored and superimposed onto bright field images. 3V: Third ventricle, OC: Optic chiasm, Scale bar: 100µm. B. Sagittal slice with vertical boxes to illustrate location of coronal slice positions. Conventions as in A. C. Phase maps illustrating peak time on the first cycle *in vitro* for each slice in A (top) and a set of SCN slices from an additional LD 12∶12 animal (bottom). Phase is depicted relative to the peak time of the whole rostral slice for each animal. D. Background-subtracted bioluminescence image of cell-like ROIs (circled in white) and raster plots of bioluminescence rhythms from cell-like ROIs, where values above and below the mean are red and blue, respectively.

SCN slices were collected from homozygous PERIOD2::luciferase knock-in mice [25] maintained under a light∶dark cycle with 12h of light and 12h of darkness (LD 12∶12). Three consecutive coronal slices corresponding to the rostral, central and caudal portions of the SCN were retained from each animal for simultaneous bioluminescence imaging (Materials and Methods, Figure 1, Video S1). Since rigorous mapping requires a consistent sectioning technique, we assessed the similarity of the bioluminescence profile of slices collected at the same position from different animals and used the bioluminescence profile to develop a metric for slice position (Figure S1). The positional metric of slices collected from different animals was similar, but not identical, which likely reflects slight variability in exact slice position and SCN morphology (Figure S1).

**Figure 2:**
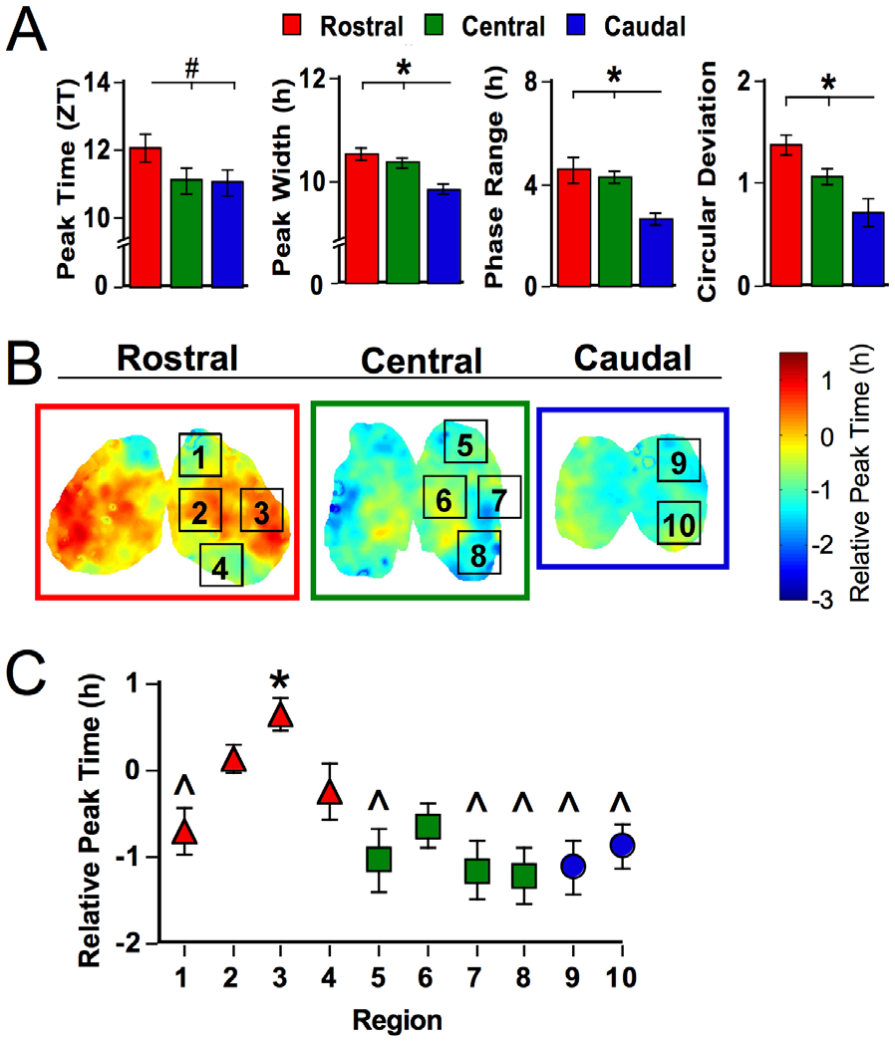
Spatiotemporal organization of SCN slices from LD 12∶12 animals. A. Rostrocaudal differences in the average phase and peak width of the field rhythm for each SCN slice and the range and circular deviation of peak phases of cell-like ROIs within each slice. # p<0.06, * p<0.01 (Tukey-Kramer HSD). B. Average phase maps illustrating regional phase differences expressed during the first cycle *in vitro*. Each slice represents the overlay of at least seven LD animals. Regions labeled were selected for analyses of cell-like ROIs, only one lobe is labeled but data from both lobes were used. C. Regional differences in relative peak time. * and ∧ indicate regions that differ from one another (Tukey-Kramer HSD).

At the level of the whole slice, rostral, central, and caudal SCN collected from LD 12∶12 animals exhibited different temporal characteristics. First, the central and caudal SCN tended to peak at an earlier ZT than the rostral slice (Figure 2A, p = 0.06). To assess the phase relationship among rostrocaudal slices and to control for between-animal variation in ZT peak time, phase of the central and caudal slices was expressed relative to the peak time of the whole rostral slice for each mouse. The relative peak time of the central and caudal SCN was ∼1h earlier than that of the rostral slice (p<0.005). Rostral, central and caudal slices also differed in the waveform of bioluminescence rhythms, with the caudal SCN expressing a smaller peak width than the rostral or central slice (Figure 2A, p<0.0005). Since peak width can be influenced by the phase relationships of underlying oscillators, we assessed the range and deviation of peak times displayed by cell-like regions of interest (ROIs) in the rostral, central, and caudal SCN (Materials and Methods). The average range of peak times for cell-like ROIs within all three SCN slices was 6.7±0.5h. Across rostrocaudal slices, there was a systematic decrease in both the range and circular deviation of peak times, with the caudal SCN displaying the smallest phase heterogeneity (Figure 2A; p<0.0005 for each test). Despite robust differences in phase and waveform, rostrocaudal slices did not differ in period length (Rostral: 23.3±0.2, Central: 23.6±0.2, Caudal: 23.4±0.1, p>0.3).

To assess the spatial organization of SCN phase heterogeneity, we generated average phase maps for each slice position (Methods and Materials, Figure 2B). Within the rostral SCN, early-peaking regions were located in both dorsal and ventral compartments, whereas late-peaking regions were located within medial and lateral compartments. In contrast, a large proportion of the central slice expressed a relatively early peak time, but a relatively late-peaking region was located within the medial compartment. Peak time across the caudal slice was largely homogeneous and early in phase. Similar regional phase relationships were observed in slices from individual animals (i.e., the caudal SCN exhibited an early peak time and the rostral SCN contained a late-peaking node), although between-animal variability was evident (Figure 1C). When the average phase of cell-like ROIs was determined for spatially segregated regions, phase heterogeneity was evident across regions (Figure 2C, p<0.0001). In particular, the lateral region within the rostral SCN exhibited a significantly later peak time than most regions within the central and caudal SCN. These regional phase differences were largely maintained across the first three cycles *in vitro*, although changes in the phase of regions within the central SCN were evident (Figure S2).

### Regional differences in period or waveform do not account for regional phase differences

Classic theories of circadian organization propose that phase heterogeneity is determined by differences in other oscillatory parameters (e.g., period); therefore we investigated the mechanistic bases of SCN spatiotemporal organization by testing whether SCN regions differed in other circadian parameters that could account for phase differences. Regional differences in period were not detected (Figure S3A, p = 0.7), and period accounted for only a minor proportion of variance in phase (r^2^ = 0.03). Peak width varied across regions (Figure S3B, p<0.03), but regional differences did not meet criteria for multiple comparisons. Further, peak width was positively correlated with phase, but accounted for only a minor proportion of variance (r^2^ = 0.03). Similarly, regional differences in amplitude, brightness, and number of cell-like ROIs were evident (Figure S3C, S3D, S3E, p<0.0001), but each variable accounted for only a minor proportion of variance (r^2^ = 0.03 for each test). Lastly, regional differences in circular deviation were detected (Figure S3F, p<0.03) but did not meet criteria for multiple comparisons, and regional circular deviation was not significantly correlated with phase (r^2^ = 0.01). Thus, regional phase differences were not linearly related to regional differences in these other parameters.

### Regional phase differences do not relate to regional differences in AVP or VIP content

To examine the relationship between regional phase heterogeneity and the distribution of AVP- and VIP-immunoreactive (-ir) cells within the SCN, we imaged and then immunolabeled rostral, central, and caudal slices from three LD 12∶12 animals (Figure S4A). Consistent with the regional phase differences described above, robust differences in peak time were evident both between and within rostrocaudal slices (Figure S4B, S4C, p<0.0001). Although not statistically significant, dorsal regions within rostral, central, and caudal slices assumed an earlier phase in this LD 12∶12 replicate (Figure S4B, S4C, p = 0.14).

The distribution of AVP-ir and VIP-ir cells was consistent with previous descriptions of the murine SCN, but many cells within the central SCN did not stain for either neuropeptide (Figure S4A). Within rostral, central, and caudal slices, the earliest regions were located dorsally and contained many AVP-ir cells. However, AVP-ir cells were found in all regions regardless of the relative phase of the region (r = −0.3, p>0.1). VIP-ir cells were contained within the ventral SCN, with the largest population located within the central slice. Like AVP-ir cells, there was no linear relationship between regional phase and the number of VIP-ir cells (r = +0.14, p>0.3).

### Regional phase differences are not driven by light input

**Figure 3:**
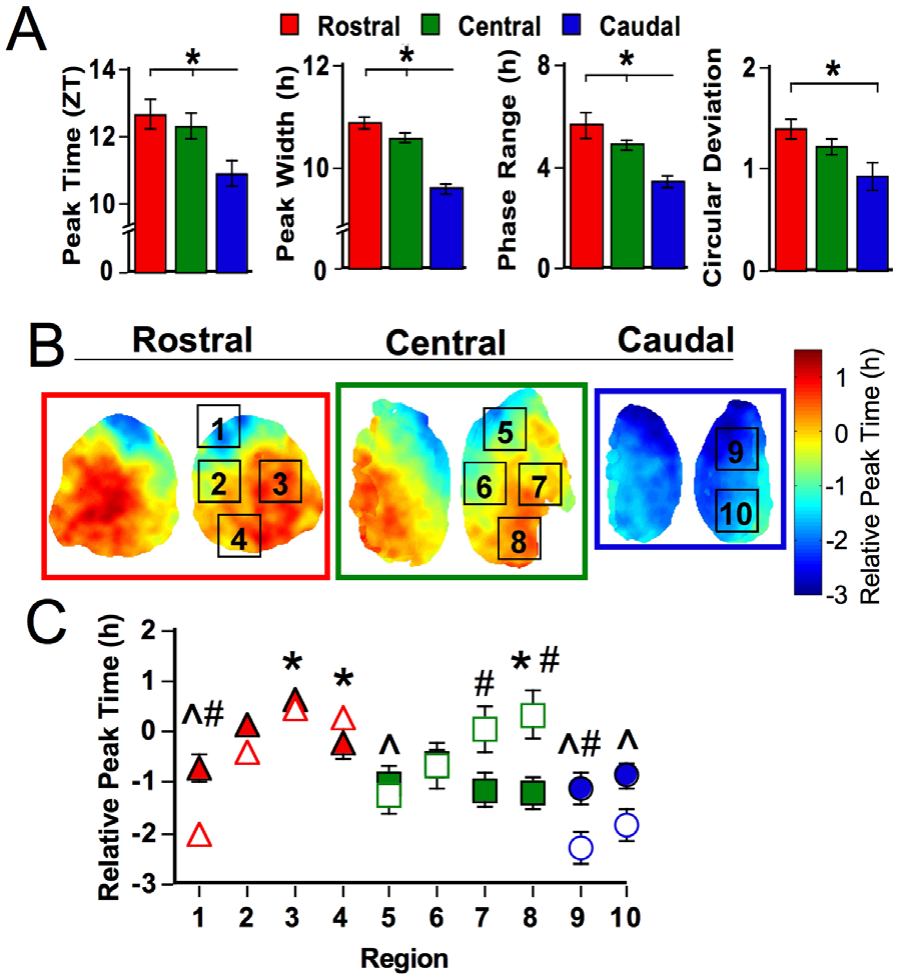
Spatiotemporal organization of SCN slices after one week in DD. A. Rostrocaudal differences in phase, peak width, range of peak times, and circular deviation of peak times. * p<0.01 (Tukey-Kramer HSD). B. Average phase maps illustrating SCN spatiotemporal organization after one week in DD. Each slice represents the overlay of at least seven DD animals. Regions labeled were selected for analyses of cell-like ROIs, only one lobe is labeled but data from both lobes were used. C. Regional differences in relative peak time across the SCN within DD slices (open symbols) compared to LD slices (filled symbols, re-plotted from Figure 2C). * and ∧ indicate DD regions that differ from one another (Tukey-Kramer HSD). # indicates regions that differ between LD and DD, p<0.005 (Least Squares Means Contrasts).

To investigate whether regional phase differences are merely driven by light input, a separate group of animals was transferred from LD 12∶12 to constant darkness (DD) for one week (Figure 3). Compared to rostral and central slices, the caudal SCN of DD animals continued to display an earlier peak time and a smaller peak width (Figure 3A, p<0.01). Unlike LD 12∶12 patterns, DD caused the central SCN to adopt a later phase that was no longer different from the rostral SCN (Figure 3A). Consistent with LD 12∶12, peak time range and deviation decreased across the rostrocaudal extent of the SCN (Figure 3A, p<0.005). The average range of peak times across DD slices was 7.1±0.6h, which did not differ from LD 12∶12 (p>0.6). Once more, rostrocaudal SCN slices did not differ in period (Rostral: 23.3±0.1, Central: 23.4±0.1, Caudal: 23.1±0.1, p>0.2).

Average phase maps and regional analyses confirmed that robust phase differences were not eliminated by one week in DD (Figure 3B, Figure 3C, p<0.0001). Overall, the pattern of regional phase differences was similar to that observed in LD 12∶12 slices, with no main effect of condition (Figure 3C, p = 0.35). However, a significant interaction between condition and regional peak time indicated that phase is modulated by release into DD in a region-specific manner (p<0.001). After one week of DD, ventral and lateral regions within the central slice adopted a later peak time relative to that displayed under LD 12∶12. Dorsal regions of the rostral and caudal slices adopted an earlier phase, which is similar to that observed in the LD 12∶12 replicate used for immunohistochemistry and indicates that the relative earliness of dorsal regions is likely influenced by a factor other than entrainment condition. Within DD slices, regional differences were evident in period, peak width, circular deviation, amplitude, and brightness (Figure S3, p<0.05 for each test); however, each of these parameters accounted for only a minor proportion of variance in relative peak time (Period: r^2^ = 0.001; Peak width: r^2^ = 0.14, Amplitude: r^2^ = 0.008, Brightness: r^2^ = 0.0005, Circular deviation: r^2^ = 0.06).

### Rostrocaudal phase differences are also evident in horizontal SCN slices

**Figure 4:**
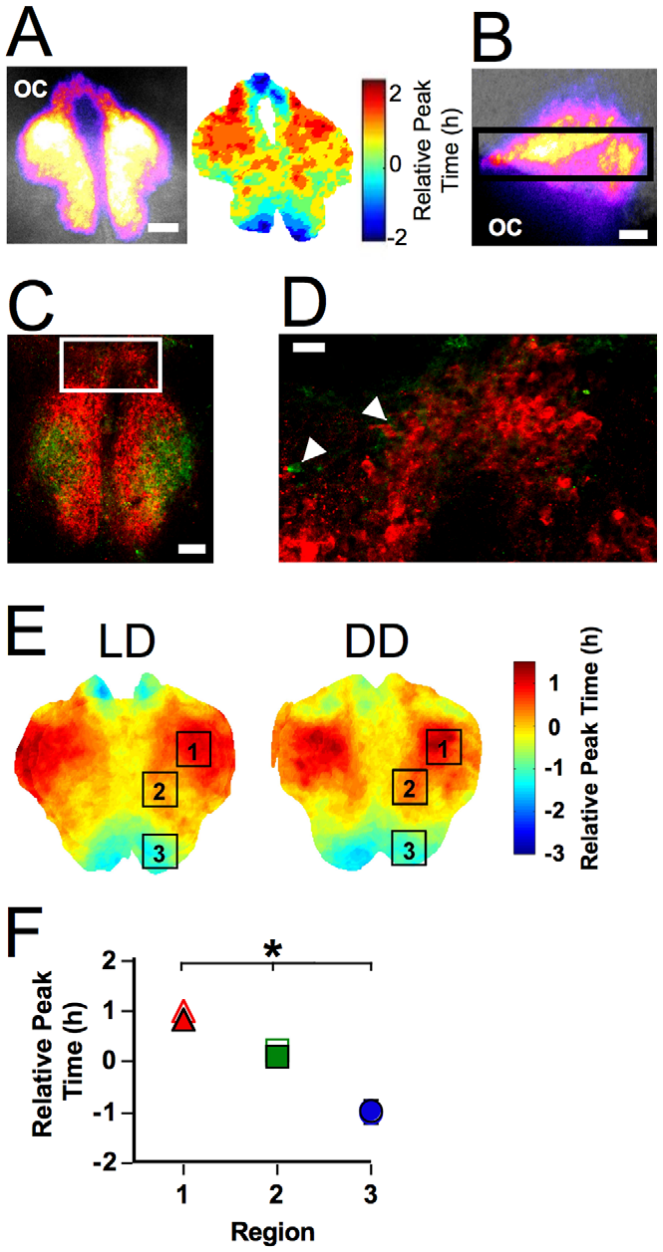
Spatiotemporal organization of horizontal SCN slices. A. Representative horizontal slice. Left: Total bioluminescence over the first 24h pseudocolored and superimposed onto the bright field image. Scale bar: 100µm. Right: Phase map illustrating peak time on the first cycle *in vitro*. Phase is relative to the peak time of the whole slice. B. Sagittal slice denoting location of horizontal slices. Conventions as in A. C–D. Immunohistochemistry for AVP (red) and VIP (green) after bioluminescence imaging illustrating that the rostrum contains a population of densely packed AVP-ir cells. Note that all frames of the z stack have been summed for images of AVP- and VIP-ir within each slice. C. AVP- and VIP-ir within a representative horizontal slice. Scale bar: 100µm. D. AVP- and VIP- ir within region indicated in C. Arrows indicate VIP-immunoreactive cells. Scale bar: 20µm. E. Average phase maps depicting regional differences in peak time on the first cycle *in vitro* within horizontal slices collected from LD 12∶12 and DD animals. F. Regional differences in relative peak time across the SCN. Regional phase did not differ between LD 12∶12 (filled symbols) and DD animals (open symbols). * regions with different phases in both LD and DD slices (Tukey-Kramer HSD).

To test whether phase differences persist when rostral and caudal regions are contained within the same slice, we next collected SCN slices in the horizontal plane (Figure 4). The bioluminescence profile of horizontal slices was in accord with previous descriptions of the SCN based on imaging in the coronal plane, with one notable exception. Below the third ventricle, the rostral-most pole of the SCN formed a thin bridge-like structure containing a densely packed group of AVP-ir neurons, which we will refer to as the rostrum (Figure 4C–D, see also [6]). VIP-ir fibers were evident across the rostrum and a small number of VIP-ir cells were detected (Figure 4C–D). Not only were robust phase differences observed in the rostrocaudal dimension of the SCN (see below), a phase gradient was also evident across the rostrum (Figure 4A, Video S2). Thus, the rostrum should be considered as a part of the SCN since it is contiguous with the SCN proper, expresses robust PER2::LUC rhythms characterized by phase heterogeneity, and contains densely-packed AVP neurons.

Robust phase differences were evident between rostral and caudal regions of horizontal SCN slices, with the caudal pole phase-leading the rostral pole in both LD 12∶12 and DD slices (Figure 4E–F, p<0.0001). There was no overall effect of entrainment condition (p>0.5), nor was there a region-specific effect of entrainment condition (p>0.8). The range in peak times displayed by cell-like ROIs within horizontal SCN slices did not differ between LD 12∶12 and DD (3.8±0.3h and 3.7±0.4, respectively; p>0.7), but the average peak time range was significantly smaller in horizontal slices than coronal slices (LD: p<0.0001; DD: p<0.001), which may reflect the fact that horizontal slices lacked dorsal- and ventral-most regions (Figure 4B). Rostrocaudal regions did not differ in period, peak width, or amplitude (p>0.1; LD versus DD: p>0.1), although rostral and central regions were brighter than the caudal SCN (p<0.02; LD versus DD: p>0.5).

## Discussion

In mammals, the function of the central pacemaker depends on coordinated activity across a heterogeneous population of neurons. Here we have developed novel analytical tools and expanded understanding of SCN function by providing comprehensive phase maps under both entrained and free-running conditions. We find regional phase differences across all three dimensions of the SCN; however, the pattern, magnitude, and complexity depend greatly on the rostrocaudal position of the slice. Differences in slice position and regional definitions may account for discrepant descriptions of SCN spatiotemporal organization across previous studies, although the influence of other potential factors cannot be discounted (e.g., different output measures). To map SCN spatiotemporal organization, we have simultaneously imaged consecutive slices collected from the same animal and designed extensive computational analyses that investigate oscillatory function across the SCN without *a priori* regional definitions, which are techniques unique to the present study. While the resulting phase maps underscore the sophistication of SCN spatiotemporal organization, the most salient features that emerge are the early phase of the caudal SCN and the late-peaking node of the rostral SCN. The consistency of these findings despite methodological differences across experiments (e.g., entrainment condition, plane of section) demonstrates the robustness of SCN phase heterogeneity.

Release into DD for one week failed to eliminate regional phase differences, which indicates that phase heterogeneity within the SCN is not merely driven by light. Moreover, robust regional phase differences persisted over time *in vitro*. Since the SCN lacks input from the environment and other brain structures when cultured in this manner, this indicates that regional phase relationships within the SCN are intrinsically regulated. This finding is consistent with previous work using organotypic SCN slices collected from neonatal mice and cultured for 2–3 weeks before imaging, in which SCN neurons display regional phase differences [3], [19] that are re-established when reset to a common phase [3]. Since phase can be influenced by the time of cull and dissection [12], [26], it is possible that regional phase differences reflect region-specific resetting produced by dissection. However, the regional phase differences described here are consistent with studies using organotypic slices and *ex vivo* studies of clock gene expression [27]–[29], which suggests that phase heterogeneity reflects the inherent organization of the SCN.

We show here that SCN phase heterogeneity is not driven by light; nevertheless, photic stimuli modulate the relative phase of SCN oscillators [30]. Previous work using the Per1-GFP mouse indicates that phase heterogeneity persists after acute release into DD, but that the phase distribution of SCN oscillators is affected [18]. With the increased spatial resolution afforded by the present approach, we also find evidence that SCN spatiotemporal organization is modulated by release into DD. Considering both LD 12∶12 replicates, the most consistent feature distinguishing LD 12∶12 and DD is that the phase of the ventral region (region 8) within the central SCN adopts a later peak time after one week of DD (Figure 3, Figure S4). It is of interest that within the ventral region of the central SCN, we find the highest concentration of VIP-ir cells (Figure S4) that are a reliable marker for the SCN region that mediates circadian responses to light [10], [27], [31] and contains several types of light-responsive cells [7], [9], [32], [33]. While region-specific modulation of phase was not evident in horizontal slices, the ability to discriminate between LD and DD may have been limited since horizontal slices lacked the ventral-most regions of the SCN. Future research investigating the plasticity of SCN spatiotemporal organization is warranted, and it would be of particular interest to examine whether other photic manipulations modulate the phase of the central SCN [30]. However, since afferent input is not restricted to the ventral SCN, it remains possible that other environmental manipulations will affect the phase of additional SCN regions. For example, the phase difference between the rostral and caudal SCN is positively correlated with day length [15], [16], [22]–[24]. Given the sophistication of SCN spatiotemporal organization and regional differences in oscillatory properties, the novel techniques we have developed provide an attractive means to investigate SCN plasticity further.

The mechanisms underlying phase heterogeneity have been theorized to involve regional differences in intracellular function and/or intercellular connectivity [3], [18], [34]. Classic theories of circadian organization propose that oscillator phase relationships are reflected in the circadian waveform of the population and that oscillators with different period lengths will adopt different phases under entrained and free-running conditions [35]. In the current study, SCN slices displayed differences in circadian waveform that were related to the degree of phase heterogeneity within the slice, which is consistent with previous research [15], [20], [36]. However, period was not statistically different among rostrocaudal slices and no substantial relationship between period and phase was evident. Regional differences in other circadian parameters were manifest in coronal slices under some, but not all, conditions, and these rhythmic parameters did not differ among rostrocaudal regions of horizontal slices. Since the phase of PER2::LUC expression was not systematically related to these other circadian parameters, this suggests that SCN phase heterogeneity is not determined by these factors but rather regulated by SCN network properties. One caveat to this interpretation is that period and waveform may be influenced by intercellular communication in the present study. However, rostral, central, and caudal slices were cultured separately, which would abolish coupling between regions in different slices. Further, dissection likely disrupts synaptic connections between regions within a slice, although regions could be coupled effectively through a variety of mechanisms [5]. Thus, it remains possible that early- and late-peaking neurons within the same slice have different *intrinsic* properties, which can be tested under conditions that manipulate intercellular communication.

We also found little correspondence between regional phase and the presence of AVP or VIP. AVP and VIP constitute ∼20% and ∼10% of SCN cell types, respectively [6] and represent a minority of Per1-GFP positive cells [21]. Since the SCN contains numerous cell types, it is possible that SCN spatiotemporal organization is related to the presence of a neuropeptide not included here [6], [7]. It will be of interest to compare the distribution of phases displayed by SCN cells that contain AVP, VIP, and other neuropeptides. Although it is technically challenging to map bioluminescence and immunohistochemistry on a cell-by-cell basis, we are developing ways to facilitate this mapping and extending our analyses to include other SCN cell types. Incorporating a cell-based approach may reveal a relationship between phase and neuropeptide content; however, it may be overly simplistic to expect that early- and late-peaking regions will be distinguished by a single neuropeptide. Rather, regional phase differences may reflect the diversity of cell types within each region and/or differences in intercellular signaling [37]–[39]. Consistent with this hypothesis, stable phase differences among SCN neurons within organotypic slices are disrupted when intercellular communication is inhibited [3], and the capacity to oscillate in the absence of intercellular communication does not correspond to a distinct subclass of SCN neurons [40].

Here we were primarily concerned with mapping phase heterogeneity, but the novel analyses developed presently can be modified to map other oscillatory parameters. For example, the core SCN has been described to have low amplitude and/or undetectable rhythms in clock gene expression and electrical activity [7], [34], [41], [42]. Consistent with these studies, we find a region within the central SCN that is less bright than medial regions within the same slice (see Figure 1A, S4) and contained cells with lower amplitude rhythms (see Figure S3). Thus, we are able to detect the phase of regions across the SCN despite the fact that all regions do not display equally high levels of PER2::LUC expression. We did not detect a robust relationship between relative phase and the number of cell-like ROIs within a region or the brightness and amplitude of PER2::LUC rhythms displayed by cell-like ROIs; however, previous research suggests that strength of clock gene expression is related to functional differences in electrophysiological properties [43].

While there is compelling evidence indicating that SCN oscillators do not necessarily adopt a common phase, the function of SCN phase heterogeneity remains unknown. The degree of phase heterogeneity in the SCN likely has functional consequences for output signals transmitted to downstream oscillators since *per1* activity correlates with AVP release [27] and electrophysiological spike frequency [18] but see [43]. Regional phase heterogeneity within the SCN may provide specifically phased output signals to different downstream tissues [13], [44]–[46]; however, the regions shown here to adopt different phases may communicate similar information to the same target tissue. Redundant signaling may augment the robustness and resilience of the system since the wider peak phase at the population level could increase the likelihood that downstream tissues will respond to output signals. Given the sophistication of SCN spatiotemporal organization and regional differences in oscillatory properties and robustness, the novel techniques we have developed provide attractive means to investigate these issues further.

### Summary

Here we have developed novel analytical tools to provide comprehensive maps of regional phase differences and to explore the underlying mechanisms. Regional phase differences across all three dimensions of the SCN are intrinsically regulated and not merely driven by light input, but influenced by photic stimuli in a region-specific manner. The present results are consistent with the hypotheses that intercellular communication and photic stimuli are key factors regulating SCN phase heterogeneity. While the intrinsic properties of an individual SCN neuron are theorized to influence its relative phase, we find that phase heterogeneity is not linearly related to regional differences in other circadian parameters or differential expression of AVP and VIP. The bilateral symmetry and consistency of SCN spatiotemporal organization across individuals and planes of section indicates that the precise phasing of oscillators within the SCN is a robust feature important for pacemaker function. Future studies using these novel analytical tools to investigate the plasticity of SCN spatiotemporal organization may provide further insight into the bases and function of regional phase differences.

## Materials and Methods

### Animals and Housing

These studies were approved by the Institutional Animals Care and Use Committee of the Morehouse School of Medicine (protocol # 09-30) and conformed to the Guide for the Care and Use of Laboratory Animals of the National Institutes of Health. All efforts were made to minimize the number of animals used, and their suffering. Male and female PERIOD2::luciferase knock-in mice (5±2.4 months) were transferred to individual wheel-running cages and maintained under LD 12∶12 (lights-on: 0700 EST, photophase: 200–400 lux) or constant darkness (DD) for one week. LD 12∶12 animals were sacrificed between Zeitgeber Times 10–12 (ZT10–12, where ZT12 = lights-off) since dissections during late subjective day do not reset the SCN [12], [26]. DD animals were sacrificed between Circadian Times 10–12 (CT10–12, where CT12 = onset of locomotor activity), which was computed via linear regression fit to activity onsets on the five days before sacrifice (Clocklab, Actimetrics).

### Multi-Position Automated Bioluminescence Imaging of the SCN

After decapitation, brains were removed and sectioned in 4°C HBSS supplemented with 10mM HEPES, 2% B27, and 2.5mL/L penicillin/streptomycin using a motorized vibratome (Campden Instruments, Lafayette, IN). Three consecutive coronal SCN slices (125µm) were collected from animals in LD 12∶12 (N = 10) or DD (N = 9). SCN slices were also collected in the horizontal plane (200µm) from animals in LD 12∶12 (N = 8) or DD (N = 6). Each slice was trimmed near the edges of the SCN and cultured on a membrane (Millicell-CM, Millipore) in a sealed dish containing 1.2mL of Dulbecco's modified Eagle's medium supplemented with 10mM HEPES, 2% B27, 2.5mL penicillin/streptomycin, and 0.1mM luciferin (Molecular Imaging Products, Bend, OR).

Up to four slices were imaged simultaneously for at least 4 days (Video S1). Automated imaging of bioluminescence rhythms utilized a Zeiss Axiovert 200m or a Zeiss AxioObserver Z1 microscope with a 10× Fluar objective lens, Marzhauzer scanning stage with LUDL Mac 5000 controller, and a Stanford Photonics XR Mega 10Z cooled intensified charge-couple device camera, all housed inside a custom-built, light-tight chamber heated to 37°C. Integrated camera and microscope control was managed with Piper software (version 2.3.15 or version 2.4.7; Stanford Photonics, Palo Alto, CA). Images (1.4k×1k 16-bit) were collected at 15 frames/sec, filtered in real-time to eliminate single-image noise events (i.e., cosmic rays), and stored as 2min-summed images collected once every 10min. A 2h moving average was then applied (Piper Software), images were converted to 8-bit, pixel dimensions were reduced in half, and three consecutive images were summed to produce a series of 30min images (ImageJ Software).

### Matlab-Based Computational Analyses

For individual phase maps, a time series was generated for each 12-pixel diameter ROI on a uniform grid with 2-pixel spacing. The time series for each ROI was judged to exhibit a significant circadian rhythm if the autocorrelation coefficient with a lag of 24h was significant at α = 0.05, a local maximum occurred in the autocorrelation corresponding to a lag between 18h and 30h, and the signal-to-noise ratio of the time series was at least 1. The linear trend was eliminated and a Butterworth filter was applied once forward and once backward to remove high- and low-frequency interference, in accordance with [47]. For composite phase maps, a representative slice was selected for each coronal position and other slices collected at that position were aligned to that sample by minimizing the sum of squared difference of the 24h-summed bioluminescence profiles. Time of peak luminescence was averaged at each aligned ROI where at least 7 slices (coronal SCN) or 4 slices (horizontal SCN) were judged to be significantly rhythmic. Using composite phase maps, spatially segregated regions with different relative phases were selected for analyses of cell-like ROIs.

An iterative process was employed to locate and extract data from cell-like ROIs after background and local noise subtraction (Figure 1D, S5). To remove non-SCN background, images were summed over the second cycle of the field rhythm and the summed brightness value corresponding to the 70th percentile was subtracted from each pixel. Any resulting negative values were set to zero. Local background noise produced by neighboring cells was removed from each pixel by subtracting the 50th percentile brightness value within a 7×7 pixel neighborhood. For each iteration of the cell-picking process, the brightest pixel was located in the image and classified as a rhythmic cell-like ROI if the pixel was located within a cluster of at least 10 bright pixels, the 48h time series correlated with a 24h-period sinusoidal curve, and the 96h time series had three clear peaks. To remove the contribution of that cell-like ROI in the next iteration, a Gaussian template centered at that location was subtracted from the image. This process was repeated until no locations remained that passed the required tests. Inspection of Matlab-extracted cell-like ROIs by two trained observers yielded >85% agreement between hand-picked and Matlab-picked cells (n = 6 slices). Moreover, inspection of bioluminescence rhythms from cell-like ROIs verified that PER2::LUC rhythms were not bimodal or ultradian, which would have indicated the presence of multiple cells rather than a single cell (Figure 1D, S5).

### Immunohistochemistry

Rostral, central, and caudal SCN slices (150µm) were collected at ZT10 from a separate group of LD 12∶12 animals (N = 3) and processed for AVP and VIP. SCN slices were collected at 150µm for this experiment since slightly thicker slices withstood removal from the membrane and processing. After 48h of imaging, SCN slices were cultured with colchicine-treated medium (25µg/ml) for 24h at 37°C, fixed with 4% paraformaldehyde for 24h, and then removed from the membrane before being immersed in 10%, 20%, and 30% sucrose solutions for 24h each. Slices were transferred to wells as free-floating sections, rinsed in 0.1M phosphate buffer solution (PB), then incubated for 48h at 4°C in primary antibodies (Anti-AVP raised in guinea pig; Anti-VIP raised in rabbit; Bachem, Torrance, CA) diluted (1∶1K, 1∶500, respectively) in PB+2.5% normal donkey serum+0.3% TritonX-100. Slices were rinsed in PB before a 2h incubation at room temperature in secondary antibodies (Dylight 594 anti-guinea pig, Dylight 488 anti-rabbit; Jackson ImmunoResearch, West Grove, PA) diluted (1∶200) in PB+2.5% normal donkey serum+0.3% TritonX-100. Slices were rinsed in PB, mounted onto microscope slides, embedded in mounting medium (Prolong Gold AntiFade, Invitrogen, Carlsbad, CA), and coverslipped before AVP- and VIP-immunoreactive (-ir) cells were manually counted using an Olympus confocal laser scanning microscope (FLUOVIEW FV300). Each SCN region was magnified for cell counting, and AVP- and VIP-ir cells were counted if present at that location across at least three consecutive frames of the confocal z stack.

### Data Analyses

Rhythmic parameters were calculated for each slice and cell-like ROI using a variety of Matlab-based scripts. Phase was defined as the peak time of the detrended time series on the first cycle *in vitro*, while average phase was taken as the sample mean direction 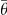 (radians) converted to hours (by multiplying by 12/π). As in [48], we defined sample mean direction 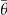, mean resultant length 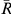, and circular standard deviation *v* via the formulas 
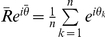
 (where θ_k_ are sample phases in radians) and 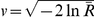. Average circular standard deviation in phase was calculated for each slice using 30 randomly selected groups of 100 cell-like ROIs and for each region using all cell-like ROIs within that location. Period was measured by the time difference between the first and second peak *in vitro*. Peak width was measured by the temporal difference between the falling and rising midpoints of peak bioluminescence on the first cycle *in vitro*. Amplitude was defined as the difference between peak and trough values of the detrended and smoothed time series during the first cycle *in vitro*. Matlab-extracted measures of rhythmic parameters were highly correlated with those extracted by hand with Lumicycle software or Excel for both whole slices (Peak time: r = 0.96, p<0.0001; Period: r = 0.79, p<0.0001; Peak Width: r = 0.6, p<0.0005) and cell-like ROIs (Peak time: r = 0.93, p<0.0001; Period: r = 0.79, p<0.0001; Peak Width: r = 0.6, p<0.0001).

Statistical analyses were performed with JMP software. Males and females did not differ statistically in the rhythmic measures studied here, and thus, data from both sexes were combined for statistical analyses. For regional analyses, the regional average of cell-like ROIs was calculated for each animal and this average was used in statistical tests to avoid spurious results produced by the large sample sizes yielded by combining data from different animals (Number of cell-like ROIs obtained in LD slices: 2876, in DD slices: 3838). Less conservative statistical tests yielded similar conclusions as those presented here. Data in figures and text are mean ± SEM.

## Supporting Information

Figure S1 **Positional analyses of bioluminescence profiles of slices collected at the same coronal position.** A. Overlay of bioluminescence profiles from 125µm slices collected from LD 12∶12 animals illustrates the consistency in the bioluminescence profiles of slices collected at each coronal position. Note that warmer colors indicate a greater number of slices that contain bioluminescent tissue within that region. B. Slice position did not systematically vary across experiments. The width-height ratio of the bioluminescence profile for rostral, central, and caudal slices collected from LD 12∶12 and DD animals varied systematically among rostrocaudal slices (p<0.0001), but not among experiments (p = 0.67). The caudal slices used for immunostaining (LD-ICC) differed from caudal slices obtained in other experiments (p<0.05), which was expected since LD-ICC slices were slightly thicker (150µm) than LD and DD slices (125µm).
(TIF)

Figure S2 **Regional phase differences over time *in vitro*.** Average phase maps illustrating regional phase differences expressed during the first three cycles *in vitro*. Each slice represents the overlay of at least seven LD 12∶12 animals. Phase is depicted relative to the peak time of the whole rostral slice on that particular cycle.
(TIF)

Figure S3 **Regional measures of period, peak width, amplitude, brightness, and circular deviation in LD 12∶12 (filled symbols) and DD (open symbols).** A. Regional period measures (LD: p = 0.7; DD: p<0.005). B. Regional peak width measures (LD: p = 0.03; DD: p<0.0001). C. Regional amplitude measures (LD: p = 0.1; DD: p = 0.008). Note the overall decrease in amplitude for DD slices (p<0.0001). D. Regional brightness measures (LD: p = 0.0001; DD: p = 0.001). Note the overall decrease in peak brightness for DD slices (p<0.0001). E. Number of Cell-like ROIs detected in each region (LD: p<0.0001, DD: p<0.0001). F. Regional circular deviation measures (LD: p = 0.03; DD: p = 0.01). Symbols along x-axis illustrate regions that differ (Tukey-Kramer HSD). LD: % versus #, DD: * versus ∧.
(TIF)

Figure S4 **Spatiotemporal organization of SCN slices processed for AVP- and VIP-ir after bioluminescence imaging.** A. Rostral, central, and caudal slices from a representative LD 12∶12. Top row: Total bioluminescence over the first 24h pseudocolored and superimposed onto bright field images for rostral, central, and caudal slices collected from the same animal. 3V: Third ventricle, OC: Optic chiasm, Scale bar: 100µm. Bottom row: AVP- and VIP-ir of these same slices. Note that all frames of the z stack have been summed for images of AVP- and VIP-ir within each slice. B. Spatiotemporal organization in SCN slices collected from LD 12∶12 animals, imaged for 2 days, and stained for AVP and VIP. Average phase maps for rostral, central, and caudal slices illustrating regional phase differences expressed during the first cycle *in vitro*. Each slice represents the overlay of all 3 animals. Regions labeled were selected for further analyses, only one lobe is labeled but data from both lobes were used. C. Regional differences in relative peak time in LD 12∶12 SCN slices used for immunohistochemistry (open symbols) compared to the first LD 12∶12 dataset (filled symbols, replotted from Figure 2C). Differences in relative peak time were evident between regions of LD 12∶12 slices used for immunohistochemistry (p<0.0001). Post hoc tests: * and ∧ indicate regions within the second set of LD 12∶12 slices that differ from one another (Tukey-Kramer HSD).
(TIF)

Figure S5 **Representative central slice collected from a LD 12∶12 animal illustrating morphology and rhythms from cell-like ROIs.** A. Background-subtracted bioluminescence image of cell-like ROIs. B. Bioluminescence rhythms from cell-like ROIs (labeled in A). Number in parenthesis indicates relative peak time for each cell-like ROI.
(TIF)

Video S1 **PER2::LUC rhythms of rostral, central, and caudal SCN slices collected in the coronal plane from a single animal.** A time-lapse movie (30min/frame) of bioluminescence rhythms during the course of a 4-day experiment.
(MOV)

Video S2 **PER2::LUC rhythms of the SCN collected in the horizontal plane.** A time-lapse movie (30min/frame) of bioluminescence rhythms during the course of a 5-day experiment. Note the gradients in PER2::LUC expression exhibited across both the rostrum and SCN proper during the peak of each cycle.
(MOV)
