# Supplementary figures and images for: Intrinsic Regulation of Spatiotemporal Organization within the Suprachiasmatic Nucleus
Source: PLoS One. 2011 Jan 7;6(1):e15869. doi: 10.1371/journal.pone.0015869 (PMC3017566; doi:10.1371/journal.pone.0015869)

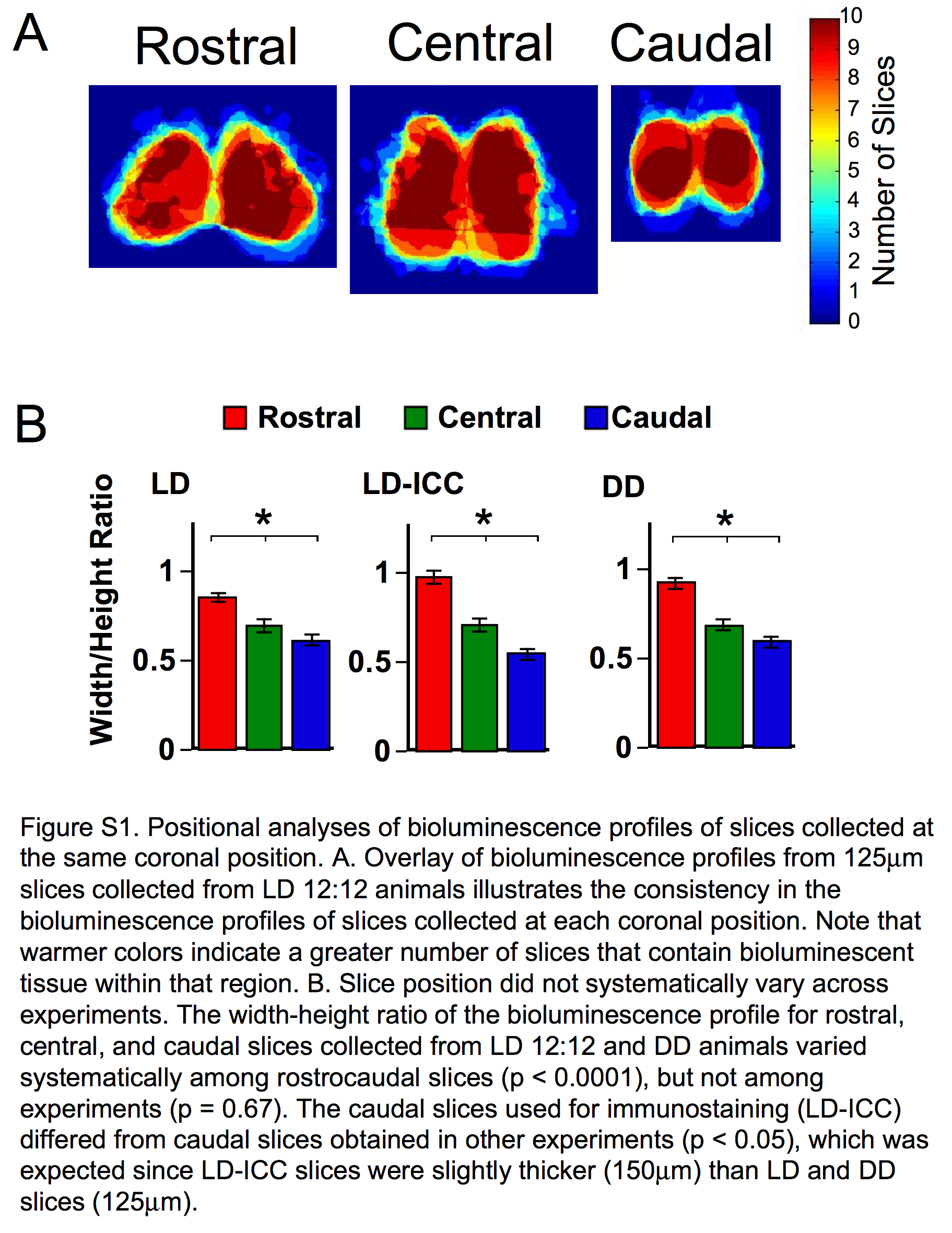

Supplement: Figure S1 — Positional analyses of bioluminescence profiles of slices collected at the same coronal position. A. Overlay of bioluminescence profiles from 125µm slices collected from LD 12∶12 animals illustrates the consistency in the bioluminescence profiles of slices collected at each coronal position. Note that warmer colors indicate a greater number of slices that contain bioluminescent tissue within that region. B. Slice position did not systematically vary across experiments. The width-height ratio of the bioluminescence profile for rostral, central, and caudal slices collected from LD 12∶12 and DD animals varied systematically among rostrocaudal slices (p<0.0001), but not among experiments (p = 0.67). The caudal slices used for immunostaining (LD-ICC) differed from caudal slices obtained in other experiments (p<0.05), which was expected since LD-ICC slices were slightly thicker (150µm) than LD and DD slices (125µm). (TIF) [file pone.0015869.s001.tif]

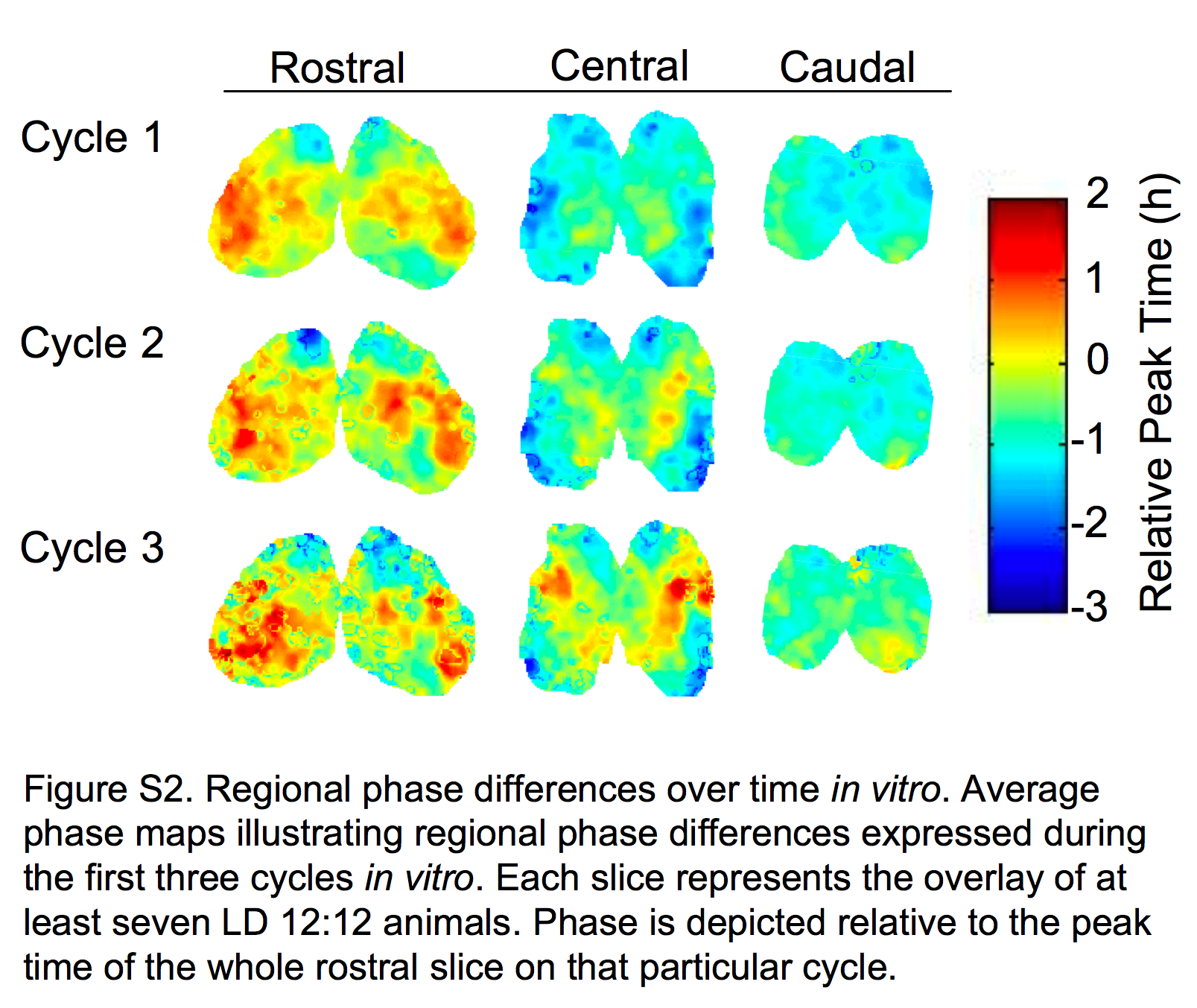

Supplement: Figure S2 — Regional phase differences over time in vitro. Average phase maps illustrating regional phase differences expressed during the first three cycles in vitro. Each slice represents the overlay of at least seven LD 12∶12 animals. Phase is depicted relative to the peak time of the whole rostral slice on that particular cycle. (TIF) [file pone.0015869.s002.tif]

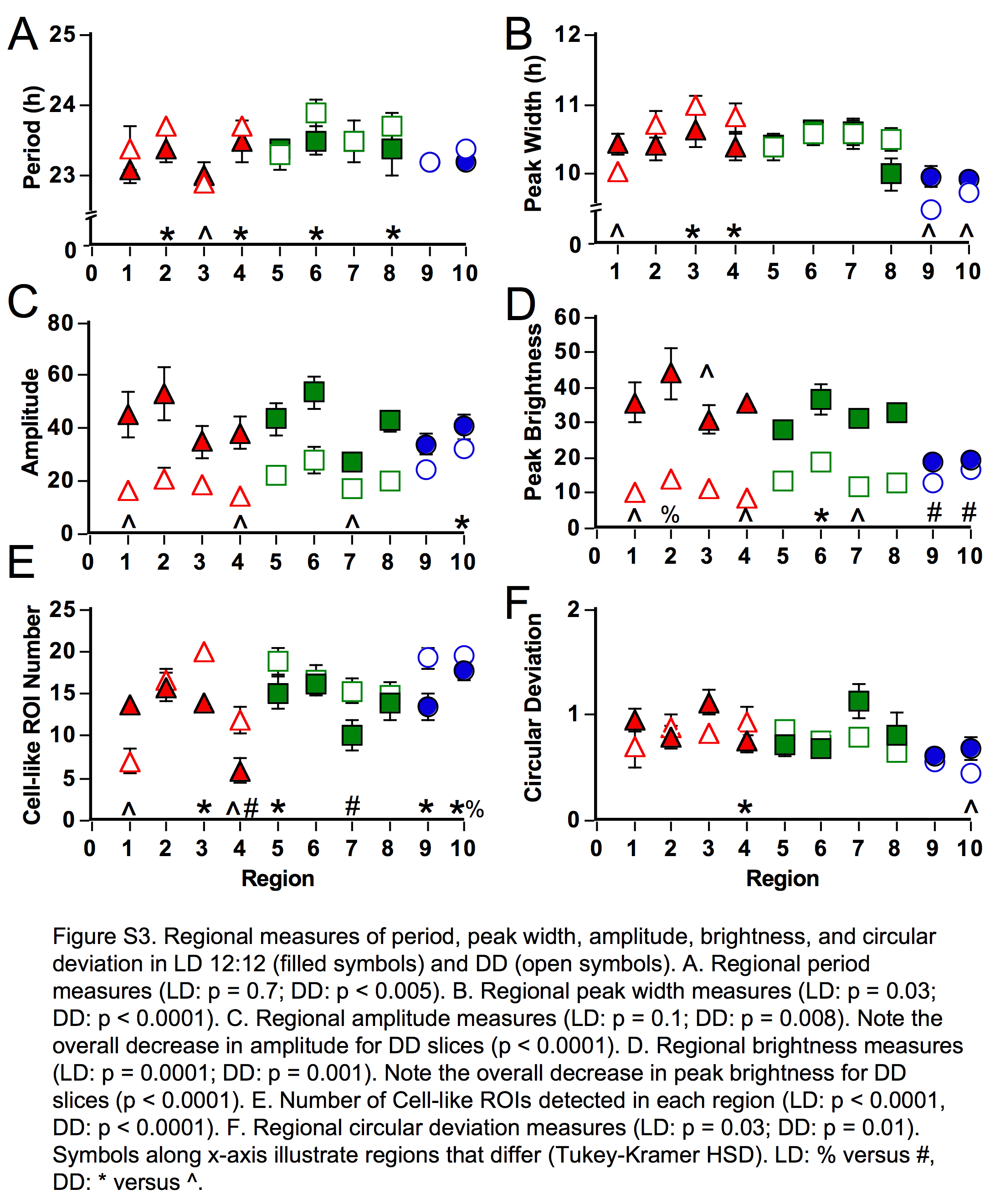

Supplement: Figure S3 — Regional measures of period, peak width, amplitude, brightness, and circular deviation in LD 12∶12 (filled symbols) and DD (open symbols). A. Regional period measures (LD: p = 0.7; DD: p<0.005). B. Regional peak width measures (LD: p = 0.03; DD: p<0.0001). C. Regional amplitude measures (LD: p = 0.1; DD: p = 0.008). Note the overall decrease in amplitude for DD slices (p<0.0001). D. Regional brightness measures (LD: p = 0.0001; DD: p = 0.001). Note the overall decrease in peak brightness for DD slices (p<0.0001). E. Number of Cell-like ROIs detected in each region (LD: p<0.0001, DD: p<0.0001). F. Regional circular deviation measures (LD: p = 0.03; DD: p = 0.01). Symbols along x-axis illustrate regions that differ (Tukey-Kramer HSD). LD: % versus #, DD: * versus ∧. (TIF) [file pone.0015869.s003.tif]

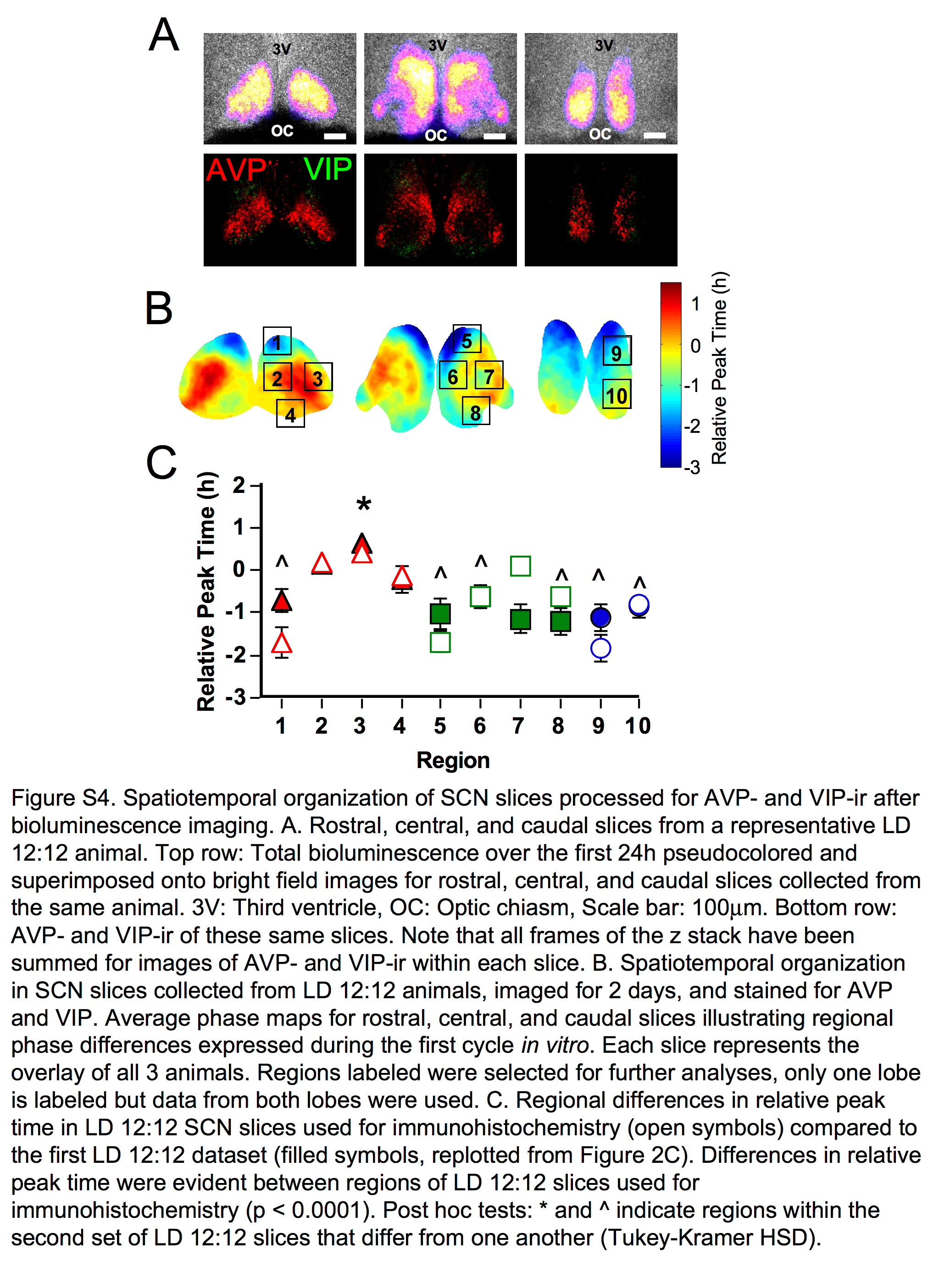

Supplement: Figure S4 — Spatiotemporal organization of SCN slices processed for AVP- and VIP-ir after bioluminescence imaging. A. Rostral, central, and caudal slices from a representative LD 12∶12. Top row: Total bioluminescence over the first 24h pseudocolored and superimposed onto bright field images for rostral, central, and caudal slices collected from the same animal. 3V: Third ventricle, OC: Optic chiasm, Scale bar: 100µm. Bottom row: AVP- and VIP-ir of these same slices. Note that all frames of the z stack have been summed for images of AVP- and VIP-ir within each slice. B. Spatiotemporal organization in SCN slices collected from LD 12∶12 animals, imaged for 2 days, and stained for AVP and VIP. Average phase maps for rostral, central, and caudal slices illustrating regional phase differences expressed during the first cycle in vitro. Each slice represents the overlay of all 3 animals. Regions labeled were selected for further analyses, only one lobe is labeled but data from both lobes were used. C. Regional differences in relative peak time in LD 12∶12 SCN slices used for immunohistochemistry (open symbols) compared to the first LD 12∶12 dataset (filled symbols, replotted from Figure 2C). Differences in relative peak time were evident between regions of LD 12∶12 slices used for immunohistochemistry (p<0.0001). Post hoc tests: * and ∧ indicate regions within the second set of LD 12∶12 slices that differ from one another (Tukey-Kramer HSD). (TIF) [file pone.0015869.s004.tif]

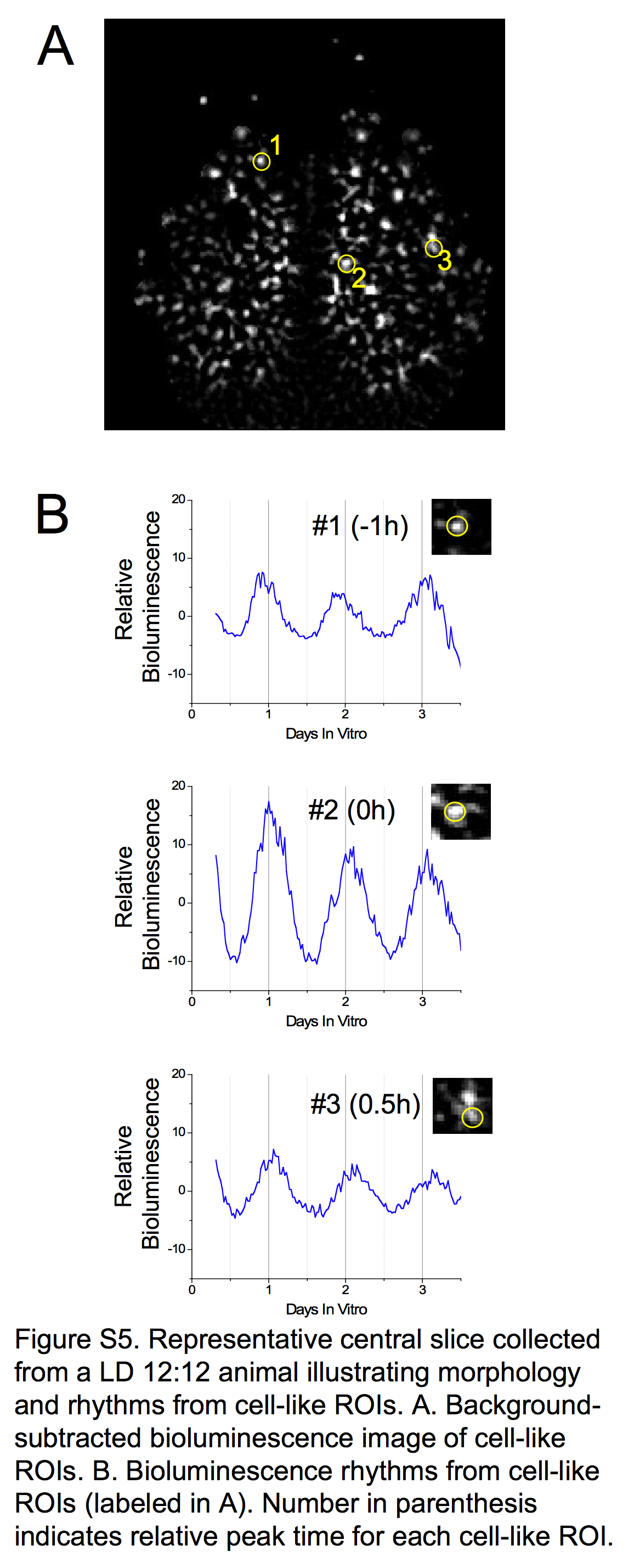

Supplement: Figure S5 — Representative central slice collected from a LD 12∶12 animal illustrating morphology and rhythms from cell-like ROIs. A. Background-subtracted bioluminescence image of cell-like ROIs. B. Bioluminescence rhythms from cell-like ROIs (labeled in A). Number in parenthesis indicates relative peak time for each cell-like ROI. (TIF) [file pone.0015869.s005.tif]
